# Review of Multi-Criteria Decision-Making Methods in Finance Using Explainable Artificial Intelligence

**DOI:** 10.3389/frai.2022.827584

**Published:** 2022-03-10

**Authors:** Jurgita Černevičienė, Audrius Kabašinskas

**Affiliations:** Department of Mathematical Modelling, Faculty of Mathematics and Natural Sciences, Kaunas University of Technology, Kaunas, Lithuania

**Keywords:** multiple criteria decision aid (MCDA), artificial intelligence, explainable artificial intelligence (XAI), interpretability, financial decision-making, investment decision-making

## Abstract

The influence of Artificial Intelligence is growing, as is the need to make it as explainable as possible. Explainability is one of the main obstacles that AI faces today on the way to more practical implementation. In practise, companies need to use models that balance interpretability and accuracy to make more effective decisions, especially in the field of finance. The main advantages of the multi-criteria decision-making principle (MCDM) in financial decision-making are the ability to structure complex evaluation tasks that allow for well-founded financial decisions, the application of quantitative and qualitative criteria in the analysis process, the possibility of transparency of evaluation and the introduction of improved, universal and practical academic methods to the financial decision-making process. This article presents a review and classification of multi-criteria decision-making methods that help to achieve the goal of forthcoming research: to create artificial intelligence-based methods that are explainable, transparent, and interpretable for most investment decision-makers.

## Introduction

Artificial Intelligence (AI) has grown significantly in use and is becoming more standardised in the twenty-first century. Artificial intelligence is increasingly applied in the financial industry and is likely to become more important in the coming years. Modern applications of AI in the financial sector are also diverse and extensive, at both the front and back ends of business processes. Examples of modern artificial intelligence applications in finance include transaction data analysis, improved chatbots, identity checking during client registration, fraud detection in claims control, pricing in bond trading, anti-money laundering monitoring, price differentiation in auto insurance, automated analysis of legal articles, risk control, portfolio management, client relationship control, and execution of trade and investment transactions.

Multi-criteria methods are widely used for decision-making in various commercial and financial contexts because of the diversity of solutions they can provide. In many studies in which financial decision-making problems have been evaluated, financial decisions have been shown to be multidimensional (see, for e.g., Hallerbach and Spronk, 2002; Govindan and Jepsen, 2016; Kabašinskas et al., 2019). Hence, most scientists and practitioners apply the methods of multi-criteria operations research when solving financial decision-making problems.

Depending on the number of guidelines, decision-making can be difficult, because there can be several ways to determine whether and when to take action. In an increasingly globalised environment, the amount of data and the number of decision options and points of view that a decision-maker (DM) must take into account can increase rapidly. In addition, finance is a very competitive arena, and a wrong decision can lead to financial losses that are often not fixed.

Examples of objectives involved in financial decisions include maximising profitability, liquidity, financial value, and social return and minimising risk, costs, and environmental damage from investments. Note that on many occasions, objectives can be conflicting. Doumpos and Zopounidis (2013) highlighted recent trends in financial decision support, including new perspectives on the use of big data, analytics, new formulations, and different types of platforms for financial transactions or products (e.g., social lending and crowdfunding). The successful choice of the most suitable multi-criteria decision-making method must take into account a number of different points of view to ensure consideration of each of the important aspects of the problem and potentially the relationships between the criteria as well.

Large digital data sets are new challenges for decision-making in finance. Many classical financial econometric or optimisation models face difficulties or are difficult to interpret when applied to digital financial big data. Big financial data require improvements to classical techniques to correctly characterise the information hidden in the data, as well as modelling and forecasting techniques that take into account the possibility of rapid changes in the data. Many financial multi-criteria decision-making tasks are performed using artificial intelligence methods because such methods can often yield better performance results than conventional methods. Typically machine learning algorithms may provide better prediction results but operate with a low degree of explainability. Explainability is one of the main obstacles to more widespred implementation of AI in financial decision-making. In the last few years, some explainability indicators, such as Shapley additive explanations (SHAP values), local interpretable model–agnostic explanations (LIME values), generalised additive models (GAM), and others, have been developed that offer some solutions to the problem of explainability.

It is important to note that multi-criteria and multi-objective optimisation is understood in this article as special class of multi-criteria decision-making (MCDM) in which all decision variables are quantitative and objective functions that can be evaluated. However, in MCDM, objective functions may have no mathematically expressed form, and decision variables can be mixed (i.e., both quantitative and qualitative). MCDM has the desired properties of being transparent and maintaining auditing possibility (Dodgson et al., 2001). The development of hybrid multi-criteria decision-making/artificial intelligence (MCDM + AI) techniques could address to some degree the explainability problem of artificial intelligence. However, these two techniques are usually discussed separately in the literature. This review attempts to address this gap by reviewing a wide range of MCDM approaches in many different fields in which AI is used to solve financial problems.

The main purpose of this article is to present a review of MCDM methods that can contribute to achieving the goal of forthcoming research in creating artificial intelligence-based methods that are explainable, transparent, and interpretable for most investment decision-makers.

The remainder of this article is organised as follows. In the next section (section the challenge of developing multi-criteria decisions and methods), MCDM problems and methods are described. A definition of artificial intelligence, a review of fields in which MCDM frameworks are used, and a discussion of explanatory AI are then presented in section artificial intelligence. Examples of MCDM methods used in finance are presented in section examples of multi-criteria decision-making in finance, with a focus on classification of the methods. The relevance of the findings of this review to future research are discussed in section discussion. Finally, the article concludes with a summary of findings and suggestions for a new decision-making process for financial applications that combines MCDM and AI.

## The Challenge of Developing Multi-Criteria Decisions and Methods

Individuals and business face diverse financial challenges, including making decisions about their future pensions, loans, and investments in various funds. Many businesses, financial institutions, and financial consultants also engaged in business activities in multiple countries, including those involving collateralised debt obligations (CDOs) and mortgage-backed securities (MBSs). These are just a few examples of financial activity (Spronk et al., 2016). Multiple sources of risk, multiple policy constraints, and multiple actors are factors that indicate that financial problems are often best treated as multi-criteria decision-making problems. There three main fields of financial decision-making (Spronk et al., 2016):

1. *Capital budgeting*: Into which investment portfolio should a firm put its capital? The main problems of capital budgeting are how to evaluate capital investments, how to choose between projects that are competitive, and how to distinguish profitable projects from unprofitable ones.
2. *Corporate financing*: How should a firm's activities be financed? What contracts in the financial field should the firm sign? How many stocks should the firm issue? How much of the firm's profit should be reinvested in the company and how much should be paid as dividends? How should the creditworthiness and liquidity of the company be maintained?
3. *Financial investment*: This includes selecting a portfolio of financial securities that reflects changing consumption patterns over time.

In addition to financial risk analysis, structured financial risk management comes to the fore. In their book *Finance*, Bodie and Merton (2000) identify the three tasks of financial management as optimisation, valuation, and risk management. Other authors have characterised the main tasks of financial management as risk management, valuation, and decision-making. Regardless of which characterisation is chosen, financial management is a multidimensional decision-making problem. Historically, in operations research and management, this type of problem has been referred to as a multi-criteria decision aid (MCDA) or multi-criteria decision-making (MCDM) problem.

MCDA and MCDM involve the application of decision-making methods by financial decision-makers in cases in which it is necessary to take into account various contradictory decision-making criteria. MCDA methods have been applied to many financial problems, such as credit scoring and failure prediction (Ferreira et al., 2014; Angilella and Mazzù, 2015), portfolio selection and management (Ehrgott et al., 2004; Aouni et al., 2018), assessment of corporate performance (Bai et al., 2014), investment appraisal (Lowe et al., 2002), and choosing funds for asset investment (Kabašinskas et al., 2019, 2020).

Every multi-criteria decision-making (MCDM) process has two stages: a criteria-based evaluation of alternatives, followed by their accumulation to identify the alternative with the top aggregation score, which informs the DM's choice (Aggarwal and Fallah Tehrani, 2019). MCDM methods allow for intentional conclusions to be made, as they can deal with the inherent complexity of many issues, as well as the understanding that results from the involvement of multiple participants (De Brito and Evers, 2016).

The main advantages that MCDA models offer in financial decision-making can be summarised as follows (Zopounidis, 1999):

1. Systematisation of complex evaluation problems,
2. Completeness of the evaluation process achieved by introducing both quantitative and qualitative evaluation criteria,
3. Valuation transparency in support of financial decisions,
4. The possibility of implementing flexible, complex, realistic scientific methods in making financial decisions.

**Figure 1:**
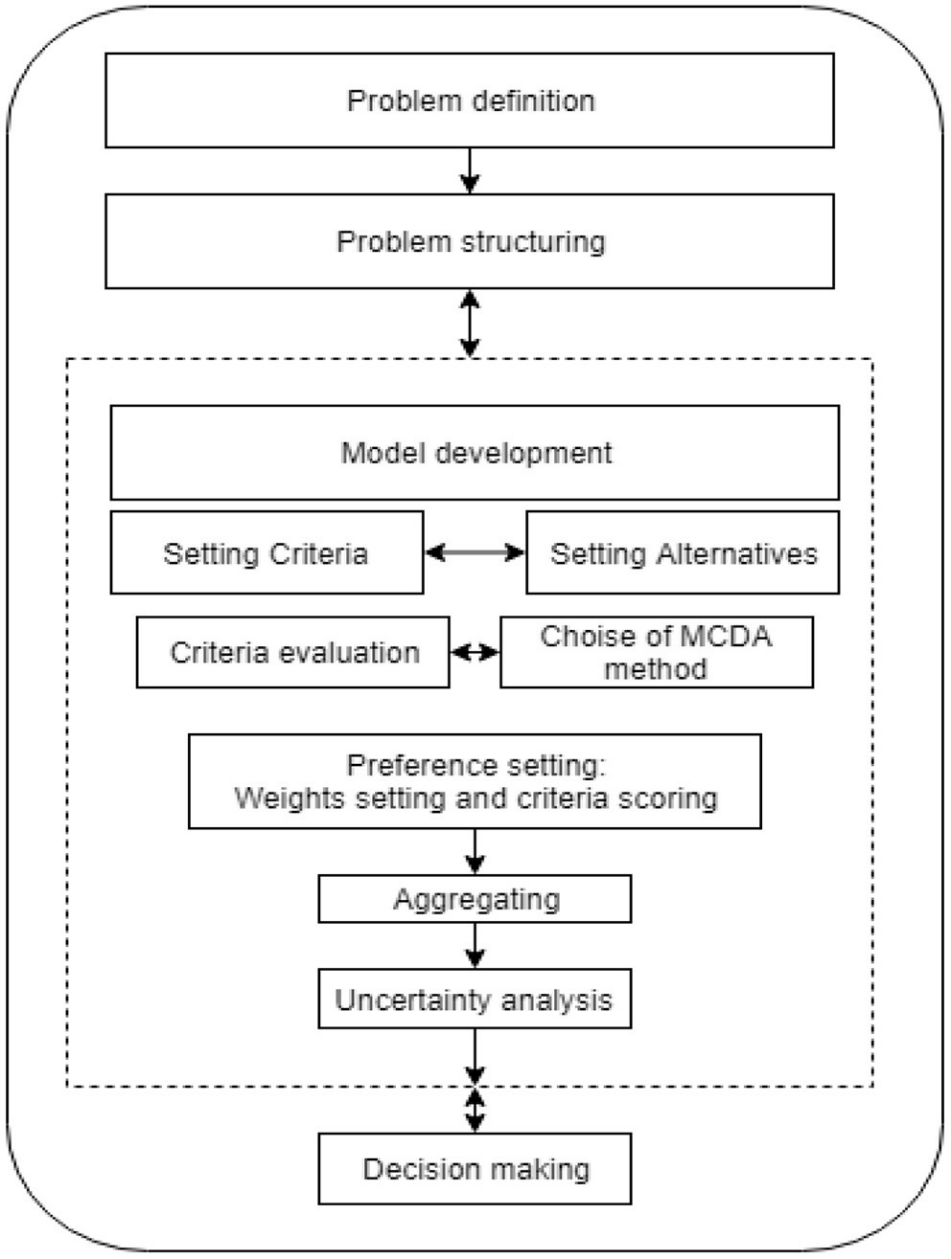
Multi-criteria decision-making process [based on Zopounidis (1999)].

As Figure 1 shows, the MCDA process begins by identifying a problem requiring a decision and identifying the key goals that need to be achieved to reach the required decision. The next step in the multi-criteria decision-making process is problem structuring, which involves identifying decision-making alternatives and the criteria against which these alternatives are to be evaluated.

Real-world applications are often treated as MCDM problems. The evaluation and selection of criteria should take into account the fact that some criteria may conflict with each other because of a lack of completeness, redundancy, reciprocity, and independence, which may complicate or confuse the decision-making process. Even in simpler cases involving only qualitative criteria, data quality can be a source of statistical vagueness. Difficulties can arise in MCDM processes not only in selecting the necessary criteria but also in quantifying the data, defining the problem, and identifying the optimal solution. Alternatives are derived from among a number of options based on prioritisation or hierarchical ranking.

As noted by Roy (1985), “the ultimate goal of the MCDM modelling framework is not to discover the best solution to a problem but to provide a method that helps an individual taking part in a decision process to shape and transform her/his preferences or to make a decision based on her/his objectives. A general MCDM problem can be expressed in the form of a (*M* × *N*) decision matrix, where *M* is the number of alternatives and *N* denotes the number of decision criteria.”

Taking into account the priorities and evaluation criteria of the DM, the main objective of the MCDA process is to identify methods for how decision criteria can be aggregated. Based on these methods, decision support models can be developed. Roy (1985) presented a common structure that encompasses all aspects of the MCDA modelling concept, beginning with specifying a set of possible alternative solutions to the problem under consideration, which can be continuous or discrete. In the first phase of the process is also defined what kind of the output of the analysis must to be. This involves choosing an appropriate “problem” solution, which may include: (a) identifying the best choice or a set of good choices, (b) ranking the choices, (c) classifying the alternatives into predetermined categories, and (d) describing the alternatives and their characteristics (Doumpos and Zopounidis, 2013).

The second step involves identifying all of the elements involved in the decision-making procedure. In the MCDA process, these elements are described as criteria. A criterion is a real function f that measures the efficiency of the alternatives with respect to each of their individual characteristics. The third step is specifying the criteria aggregation model that meets the requirements of the task. The last step includes all the necessary supporting actions required for the effective implementation of the analysis outcomes and validation of the model's recommendations.

According to Marqués et al. (2020), MCDM techniques can be divided into two groups: (1) methods based on an assumption of a theoretically infinite number of alternatives (multi-objective), and (2) methods that require assessment of a finite set of alternatives. Another taxonomy, proposed by Pardalos et al. (1995) and used in various studies is the following: (1) multi-objective mathematical programming, (2) multi-attribute utility/value theory, (3) outranking relations, and (4) preference disaggregation analysis.

## Artificial Intelligence

Stanford professor John McCarthy created the term artificial intelligence (AI) in 1955, which he described as “the science and technology of creating intelligent machines” (Rajaraman, 2014). Some researchers have argued that the nature of intelligence can be traced back to the Greeks and other philosophers of the Mediterranean (Brunette et al., 2009). The Turing Test, proposed in 1950, has also been described as the beginning of AI. The “artificial intelligence” algorithm was first used at a Dartmouth College conference in July 1956. Artificial intelligence methods were initially described as being either “top-down” methods (starting from higher-level features and actions) or “bottom-up” methods (which work in the opposite way—starting at the neural level and developing higher-level features (Brunette et al., 2009). Initially, AI was often defined as the ability of machines to understand, consider, and study in the same way as humans, but as the concept of AI has evolved over the past 60 years, it has expanded to encompass a wide variety of technologies and applications (Gao et al., 2021). Even at the present time, with so much research in AI underway, it remains difficult to provide a single definition of AI. Thus, researchers need to formulate applications of AI while generalising its essence.

To support the development of the European AI Strategy, the European Commission founded the High-level Expert Group (HLEG) on Artificial Intelligence. This group provides guidance on future policy changes and ethical, legal, and social problems associated with AI. The group's Ethical Guidelines for Robust Artificial Intelligence and its Definition of AI, which provide a general understanding of the field and its possibilities and serve as supporting documentation for the HLEG's work, are the first two HLEG results on the subject of AI (Samoili et al., 2020). The description of AI provided by the HLEG is as follows:

“Artificial intelligence (AI) systems are software (and possibly also hardware) systems designed by humans that, given a complex goal, act in the physical or digital dimension by perceiving their environment through data acquisition, interpreting the collected structured or unstructured data, reasoning on the knowledge, or processing the information, derived from this data and deciding the best action(s) to take to achieve the given goal. AI systems can either use symbolic rules or learn a numeric model, and they can also adapt their behaviour by analysing how the environment is affected by their previous actions.”

**Figure 2:**
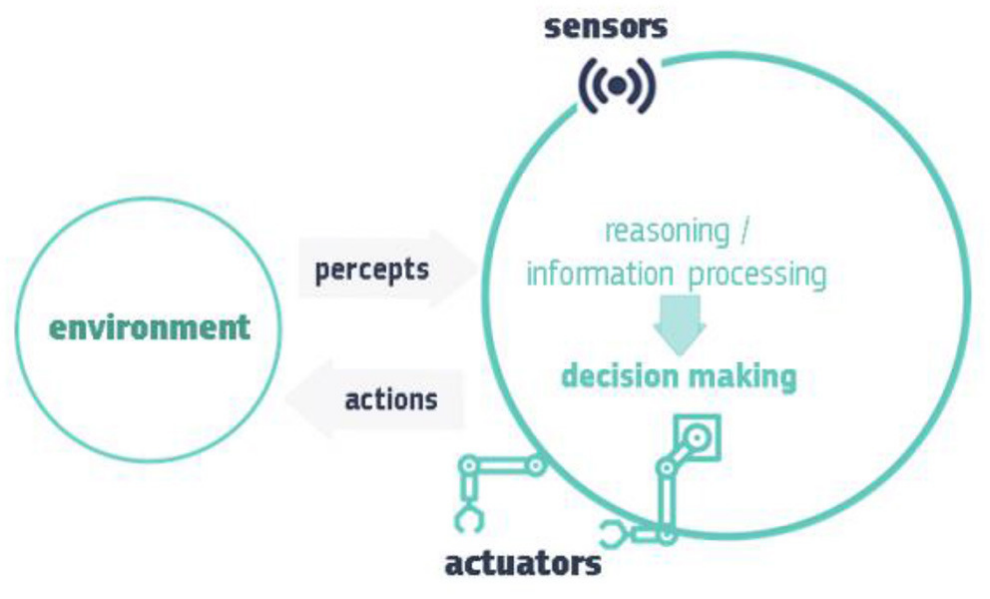
Illustration of how an AI system performs (European Commission, 2021).

The illustration of an AI system provided in Figure 2 shows how an AI system performs, i.e., by collecting and interpreting data using “sensors” that help determine the environment in which the system exists, thinking about what that environment is like or processing information based on the sensor data, deciding which action is best, and then acting accordingly, using “actuators” and thus possibly changing the environment. AI systems can use symbolic guidelines or analyse a numeric model, and they can also adapt their behaviour by studying how the surroundings are changed by their actions (European Commission, 2021).

**Figure 3:**
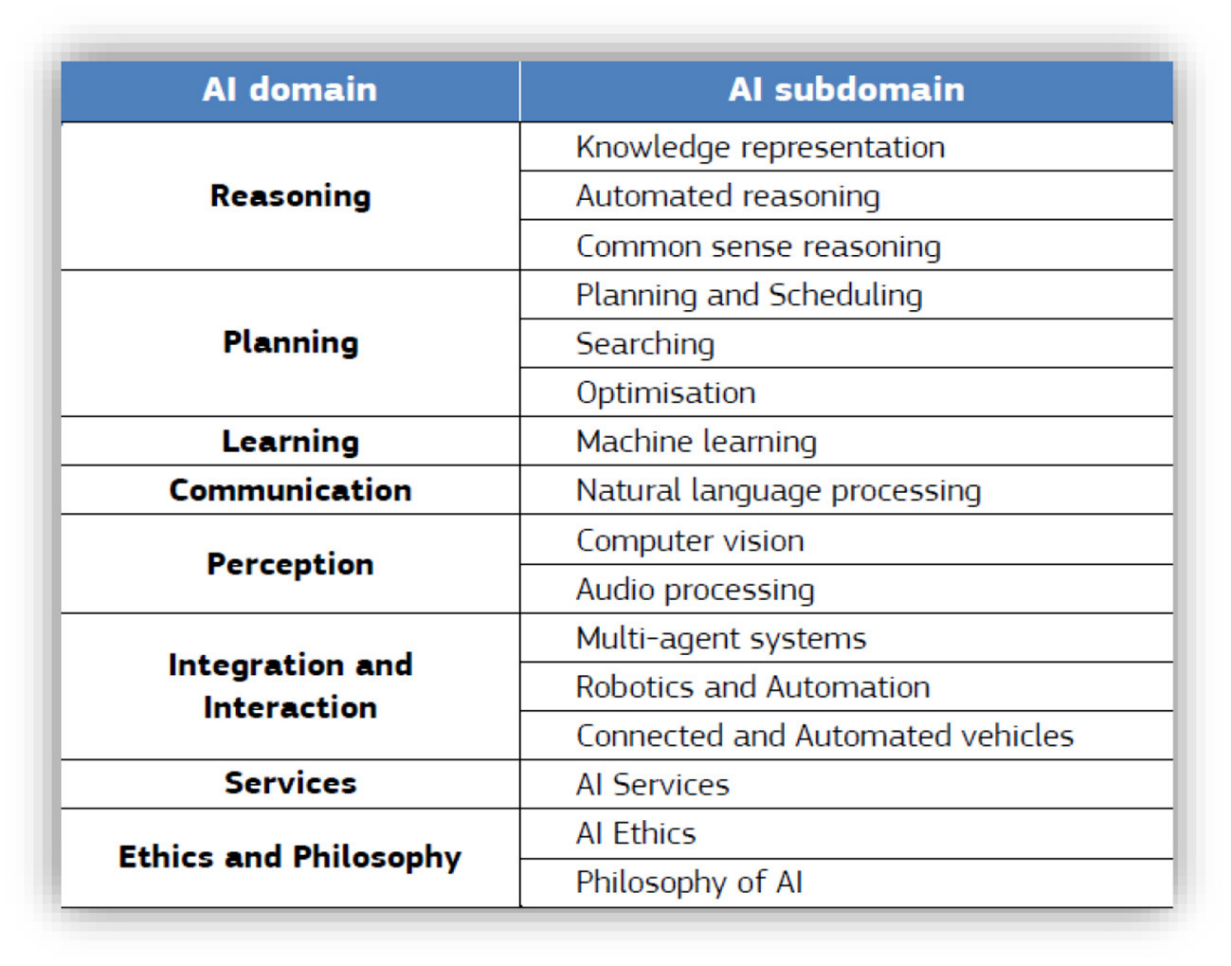
AI domains and subdomains (Samoili et al., 2020).

AI methods and subdisciplines may be divided into two classes based on their abilities: (1) reasoning and decision-making systems and (2) learning and perception. AI domains and subdomains are illustrated in Figure 3.

Artificial intelligence is steadily becoming more applicable to real life. However, the use of AI may be limited in the following five practical ways. The first limitation is the need for labelled training data. Machines don't learn by themselves in an supervised manner; they need to be taught. This means that people must spend time spent labelling and classifying the training data that AI systems require. The second limitation is that AI requires fairly large data sets. One-shot training, in which an AI model is pre-trained on a set of data and can then learn from a small number of real-world samples, is a way of reducing the need for large data sets. The third limitation is that it is frequently difficult to interpret the results of large, complex neural network systems. The fourth limitation, the complexity of generalisation, can be addressed by transfer learning, in which an AI model is trained to apply what it has learned in performing one task to learn how to perform another. A fifth limitation is the existence of biases in data and even in algorithms, which can be difficult to overcome (Bughin et al., 2018).

The growing impact of AI on the environment and society suggests that discourses, social discussions, and the use of AI technologies should be based on common principles, in line with modern management practises and social values validated by discussion and research. AI regulation sometimes assumes the meaning of law. However, the law is just one way of regulating AI (de Almeida et al., 2021). The reason that AI needs to be controlled is that mistrust exists in society concerning the many “smart” solutions offered by AI on a daily basis, including concerns about intellectual property, security, and privacy associated with a variety of medical robots, drones, autonomous vehicles, and other AI applications.

In 2021, the European Commission (EC) proposed AI regulations to the European Parliament and Council that were enacted as rules on AI and amendments to certain union laws (European Commission, 2021). The proposed regulations were the result of years of work by the commission and its supervisors, taking into account the publication of the “White Paper on Artificial Intelligence (2020). The Commission adopted an ambitious approach to AI that recognises the power of AI and its many potential benefits to society but maintains an awareness of the threats that this new technology may pose to European values and fundamental rights and principles. The EC's proposed regulation begins with identification of the following AI practises that are forbidden (European Commission, 2021):

1. Offering on the market, commissioning, or applying an artificial intelligence system that uses mental techniques outside of a human consciousness to substantially misrepresent a human behaviour in such a way as to inflict physical or psychological damage on that person;
2. AI that exploits the weaknesses of a specific institution of humans because of their age, physical or intellectual disability;
3. When AI are used by government agencies to assess or classify the reliability of individuals with a social assessment resulting in adverse or undesirable treatment unrelated to the circumstances in which the data was initially obtained, or baseless or unequal;
4. Face recognition in public places for the purpose of enforcing the law, applies differently, also subject to additional requirements, including prior authorisation for each use given by the judicial authority or an independent administrative authority in the Member State where the system exists.

The main portion of the EC's proposed AI regulation is focused on high-risk AI systems. High-risk areas identified include biometric identification and categorisation of natural persons, education and vocational training, management and operation of critical infrastructure, migration, employment, law enforcement, border control, and administration of justice and democratic processes.

### Artificial Intelligence in MCDM

Describing the theory underlying the establishment of statistical relationships is crucial for statistical learning in the data mining process and provides a basis for the necessary algorithmic procedures. The use of various types of generalised modelling forms to make algorithmic modifications in machine learning and data mining processes, especially for using big data with a large number of different types of variables, offers opportunities for MCDM (Doumpos and Zopounidis, 2013).

#### Artificial Neural Networks (ANNs)

Relying on the topology of the network and the choice of functions for the conversion of neurons, a neural network can mimic the actual functions of a complex system. This adaptability has made ANNs popular modelling tools for addressing complex problems in areas as diverse as engineering and management. This feature of ANNs has significant implications for MCDA, especially with regard to modelling the most popular frameworks. ANNs have been used successfully to study standard MCDA models derived from decision-making models with optional classification setups. Convolutional ANNs are also used in time series forecasting and clustering (Serapinaite and Kabašinskas, 2021).

#### Rule-Based Models

The machine learning research community often uses rule-based and decision tree models because the symbolic nature of such models makes them easy to understand. Most research on the use of rule-based models in MCDM focuses on the concept of rough set theory (Pawlak, 1982; Pawlak and Slowinski, 1994), which provides a comprehensive and well-proven approach to constructing decision models based on choice rules using examples. Based on the principle of the dominance relation, models of decision rules have been developed for MCDA problems using the rough set approach. Each if–then decision rule is incorporated into a set that establishes a profile that is part of the process of comparing alternatives using a dominance relationship, and part of the result is a recommended decision alternative. A decision tree is a diagram that helps to determine a path of action or show alternatives. Each branch of the decision tree represents a possible decision, and the farthest branches of the tree represent the final results of a particular decision-making process. CART, ID3, CHAID, and C4.5 are the decision tree algorithms most often used in financial classification and prediction problems. The random forest algorithm combines several decision trees with a boosting and aggregation technique, taking predictions from every tree and producing a final result that depends on the majority of the predictions.

#### Kernel Methods

*Kernel* methods are used for evaluation density, regression analysis, and pattern classification. Kernel methods using linear estimation methods place these problems in the multidimensional domain of objects, which allows the development of complex nonlinear models for forecasting and decision-making. One of the most commonly used kernel methods is the use of support vector machines (SVMs). In addition to being used to develop standard decision models, kernel methods have also been used for robust model inference detection and in the context of multi-objective optimisation (Aytug and SayIn, 2009; Yun et al., 2009) for approximation of a group of Pareto optimal solutions to complex non-linear problems. In the process of learning SVMs, multi-objective and goal programming have been used (Nakayama et al., 2005; Nakayama and Yun, 2006). Many hybrid systems based on SVMs have also been developed.

#### Fuzzy Modelling

##### Fuzzy Multi-Objective Optimisation

Fuzzy multi-objective programming problems are similar to ordinary multi-objective programming problems (i.e., optimisation of several objective functions under certain constraints). Standard multi-objective programming problems can be described as fuzzy multi-objective programming. In a fuzzy multi-objective system, all objective problems are determined using the hypothesis of the theory of fuzzy sets, by determining the fuzzy coefficients of variables and the constraints of the solutions of the objective function.

Fuzzy multi-objective programming strategies offer a framework for dealing with optimisation issues within a less strict context concerning the feel of the imposed constraints, as well as the degree of satisfaction of the DM with the compromises that may be required in meeting the constraints.

##### Fuzzy Preference Modelling

Preference modelling is a major research topic in MCDA modelling. Modelling of preferences in decision-making can be considered in the context of utility/value theory models with multiple attributes (MAVT), as well as in the context of superiority relations. Fuzzy set theory is related to the concept of the fuzzy outranking relation. Fuzzy set theory within the context of MCDA is based on the ordered weighted averaging (OWA) approach. The OWA model is a special case of the Choquet integral and is similar to a simple weighted average model. The main difference is that instead of weighing criteria, the OWA model assigns weights to the position of one criterion value relative to other values. OWA models permit modelling of different levels of compensation (Torra, 2010).

A new trend in fuzzy MCDM is its use in the linguistic environment, as real-world problems involving many decision-makers are complex, and linguistic information may be interpreted differently by different decision-makers depending on their environments. Pang et al. (2016) proposed the probabilistic linguistic term set (PLTS) to improve the understanding of linguistic information and also the description of the binary linguistic structure of multiple expert decision-makers. The researchers presented a case study of corporate investment, which is based on the proposed new VIKOR method with nested probabilistic linguistic information. Wang et al. (2021) used the VIKOR method with the nested probabilistic linguistic term set (NPLTS) to study how firms improve their investment decisions.

#### Metaheuristics

##### Evolutionary Methods and Metaheuristics in Multi-Objective Optimisation

Metaheuristics models are applicable to all types of computationally intensive multi-objective optimisation problems (MOPs). They are based on “observed optimal behaviour in nature” and make it possible to simplify complex Pareto sets. Genetic algorithms (Gas) are probably the most popular metaheuristic models. GAs are computer programs that mimic the process of evolution to solve complex optimisation problems. They use stochastic search techniques to modify original solutions (sets) using selection, conversion of changes, and crossover operators until a good solution is found.

Differential evolution (DE) was introduced by Storn and Price (1997) to solve continuous optimisation problems. Like GA, DE uses evolutionary agents to transform the generation of solutions but does so based on greedy search techniques, which ensures that solutions are firmly developed across every iteration.

A third class of techniques for solving MOPs involves such metaheuristic algorithms as simulated annealing, tabu search, colony optimisation, and particle swarm optimisation, which have been proven to be very successful in solving complex optimisation problems of a combinatorial nature.

##### Stochastic MCDM Methods and Applications

The problem of alternative selection when many variables exist in a stochastic form is called stochastic multi-criteria decision-making (SMCDM). Prospect theory, stochastic dominance, and regret theory are based on the stochasticity of the criteria. Because of its ability to accommodate high levels of ambiguity and uncertainty, SMCDM has gained considerable popularity in addressing MCDM problems in a variety of scenarios. In a detailed review, Celik et al. (2019) found that more than a quarter of the applications of SMCDM were to finance problems. These applications focus on specific issues such as credit scoring, investment project choices, enterprise selection, pension fund selection, bank investment evaluation, and even luxury car selection.

Stochastic dominance (SD) permits efficient or inefficient securities to be identified. One of the possibilities for integrating SD-based decision-making rules is the development of robo-advisory solutions to many financial problems (see Kabašinskas et al., 2020).

### Explainable AI

According to “*Explainable AI: the basics, a policy briefing*” by the The Royal Society (2019), “As AI technologies become embedded in decision-making processes, there has been discussion in research and policy communities about the extent to which individuals developing AI, or subject to an AI-enabled decision, are able to understand how the resulting decision-making system works” (The Royal Society, 2019). The reasons that some form of interpretability in AI systems may be necessary include protecting against bias, providing users with confidence that AI systems are working well, complying with policy requirements or regulatory standards, and helping developers to understand why a system works in a particular way, determine its vulnerabilities, or confirm its outputs.

Miller (2017) identified transparency and interpretability as among the most important problems with artificial learning models. According to Miller (2017), one of the benefits of improving the explainability of AI systems is increased confidence in such systems: “If users understand what led to an AI-generated decision or recommendation, they will be more confident in its outputs.”

Zopounidis (1999) noted that interpretions of decisions and results are often more important than their level of sophistication. Thus, sophisticated methods are not used primarily in practise because their outputs can be very difficult for financial decision-makers to understand.

In the design of a machine learning (ML) model, consideration of interpretability as an additional design driver can improve the model's performance, for three reasons (Arrieta et al., 2019):

- Interpretability helps to ensure fairness in decision-making. Interpretability helps to find and correct biases in the training database.
- Interpretability makes it easier to ensure reliability by highlighting possible perturbations that can change a forecast.
- Interpretability can guarantee that there is an underlying truthful causal relationship in the model's reasoning. Interpretability can thereby act as a guarantee that only significant variables are involved in producing a result.

This means that a practical explanation of the system should help in understanding the modelling and forecasting processes, recognising the rules of modelling, or assuming a possible violation of the model. According to Arrieta et al. (2019), the concept of explainable AI (XAI) proposes the creation of a set of machine learning methods that

- Create more understandable models while maintaining a high level of training efficiency (for example, predictive accuracy).
- Allow people to understand, properly trust and control a new generation of AI partners.

**Figure 4:**
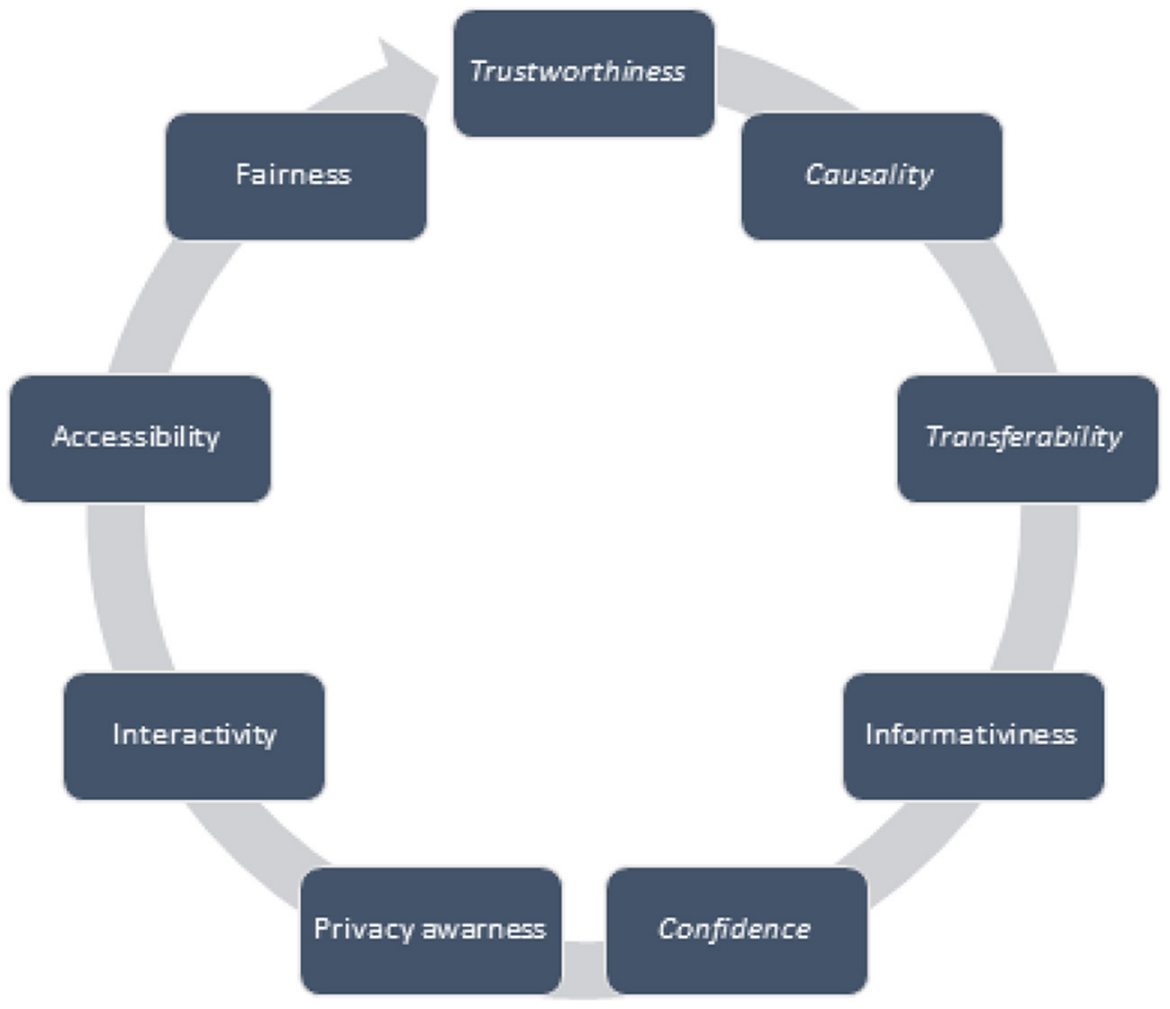
XAI goals.

The different purposes of the need for interpretation of the ML shown in Figure 4 may help to distinguish the purpose for which some clarity of the ML is provided. *Trustworthiness* can be thought of as the confidence that a model will work as intended when applied to a particular problem. Trustworthiness is a property that is not easily quantified. Another important goal in ensuring model explainability is showing *causality* between data variables. Causation includes correlation, so developing an explainable ML model may involve seeking to validate the outcomes obtained through causality inference strategies or offering the primary intuition of feasible causal relationships given the available facts (Arrieta et al., 2019). Explainability also contributes to *transferability*, as it can make it easier to identify the limits that might affect the model so that it can be better understood and implemented. The transferability of an ML model can be understood as the ability to reuse its expertise in solving another problem. *Information* about the problem under consideration should be provided using explainable ML models. Almost all rule extraction methods justify their approach to finding a simpler understanding of what the model does from the inside, claiming that the information can be presented in these more understandable examples, which they consider an explanation of the preceding. *Confidence* should always be evaluated according to the model in which trustworthiness is expected. Ways to keep confidence under control vary depending on the model. The explainable model should contain details about the confidence of its active state. Explainability should be the purpose of avoiding unfair or unethical use of the results of the algorithm. Therefore, the explainable ML model, suggests a clear visualisation of the effect-affecting relationship, which allows for ethical or *fairness* analysis of the model. *Accessibility* allows end-users to play a major role in the process of developing and implementing a specific ML model. As one of the goals of an explainable ML model is the ability of a model to be *interactive* with the end-user.

**Figure 5:**
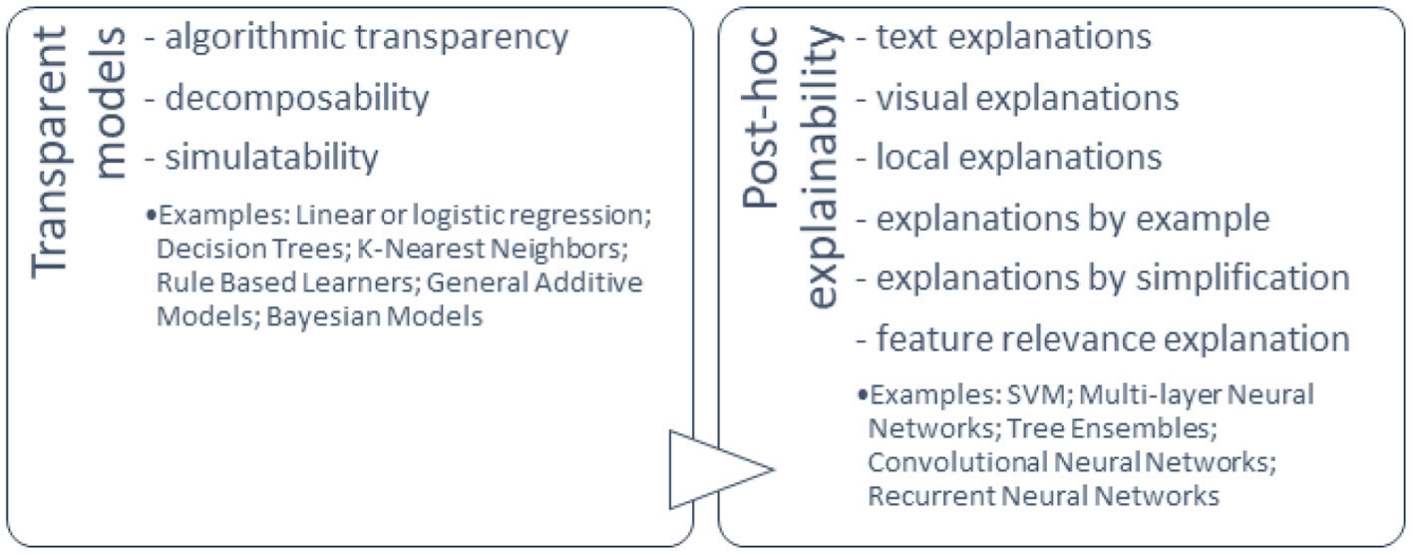
Classification of ML explainability.

Models that can be interpreted by design and those that can be explained using external XAI methods are clearly distinguished in various literature sources (Figure 5).

Doran et al. (2017) characterised three notions of explainable AI: vague systems that do not provide insight into their algorithmic mechanisms, interpretable systems that allow users to investigate their algorithmic statistics, and comprehensible systems that provide information that allow user-driven explanations of how a conclusion is reached.

There is a lack of agreement regarding the vocabulary and various definitions associated with XAI. A balance needs to be obtained between accuracy and interpretability, i.e., among the clarity of the information given by the system on its inner functioning, and the completeness of this description.

The comprehension of different observer groups does not have to be at the same level to provide insight. The same understanding of the system should be possible regardless of whether the observer is an expert in the field, a policy-maker, or a user without knowledge of machine learning.

1. The following four main methodological steps should be performed during the development and implementation of XAI (Leslie, 2019). When developing an approach to interpretability, it is necessary to take into account *contextual factors, potential impacts, and the needs of a specific subject area*. These include a deep understanding of the purpose for which an artificial intelligence model is being created, the complexity of explanations that the audience requires, and the levels of performance and interpretability of existing technologies, models, and methods.
2. *Black-box models*, such as neural networks, support vector machines, and ensemble methods, should be chosen only when their excellent modelling capabilities best match the characteristics of the problem under consideration.
3. The implementation should include *detailed specification, testing*, and *evaluation* of effective descriptive strategies and *analysis* of whether the coverage and scope of existing descriptive methods are in line with the needs of the domain and the context of the application in which the model is to be used. An interpretability action plan should be formulated that sets out the strategy for providing explanations, including a detailed time frame for the implementation of the plan, as well as defining the roles and responsibilities of the team involved in the workflow.
4. *Rethinking interpretation* in terms of a person's abilities, cognitive skills, and limitations.

These methodological principles ensure that the objective of explainability is achieved by including in the process all the different requirements of the participants, in conjunction with other global aspects of equal opportunities, such as sustainability, non-discrimination, accountability, and privacy.

#### AI Explainability Methods

Explainability methods are usually used in one of two ways. The AI models that are designed to be inherently interpretable, often because of their simplicity, i.e., generalised additive models (GAMs) (Caruana et al., 2015) or simple point systems (Jung et al., 2017; Zeng et al., 2017), are naturally explainable because they allow us to calculate the contribution of each feature to the final prediction in a segmental way, which makes it easier for people to understand the degree of influence of each feature and allows us to obtain useful information about the predictions of the model. The second group of explainability techniques provide post hoc explanations for the predictions made by compounded models. Examples of these techniques include local interpretable model-agnostic explanations (LIME) (Ribeiro et al., 2016) and Shapley additive explanations (SHAP) (Lundberg and Lee, 2017).

The SHAP value is described on the basis of the theory of cooperative games to ensure fair reward for the player (attributes) in accordance with his contribution to the common goal (AI prediction). The SHAP value enables comparison of quantitative values between different models in a model-agnostic way (Lundberg and Lee, 2017). Globally, SHAP values increase the explainability of the model by evaluating how much each variable contributes positively or negatively to the target variable. Locally, they explain why a given observation is assigned to a particular class and the contributions of variables (Ariza-Garzón et al., 2020). Some authors have suggested that the use of SHAP values is the only explainable AI approach that has been established in an economic field (Bussmann et al., 2021). Shapley-based XAI models have been used in many research studies in the field of finance (Mussard and Terraza, 2008; Ariza-Garzón et al., 2020; Bussmann et al., 2020; Giudici and Raffinetti, 2020).

Compared to alternative XAI models, the advantage of Shapley values is that they can be used to measure the contribution of each explanatory variable for each point prediction of the ML model (Lundberg and Lee, 2017). Shapley-based XAI models, because of their independence from model data, combine application flexibility with personalisation of their results (explaining any single-point prediction) (Giudici and Raffinetti, 2020; Bussmann et al., 2021).

Some authors have observed that LIME (local interpretable model-agnostic explanations) is one of the most common explainability methods associated with black-box AI models and that the model can be used to explain any classifier, irrespective of the algorithm used for predictions (Ribeiro et al., 2016; Gramegna and Giudici, 2021; Linardatos et al., 2021). To interpret individual predictions of machine learning models, LIME uses local substitute models. For each particular instance and its corresponding prediction, simulated random data are generated around the input instance for which the prediction was generated. New predictions are made for the generated instances and weighted by proximity to the input instance when using the model. This new dataset of discomposed instances is trained as a simple explainable ML model, e.g., a decision tree or regression model. The original black-box model is therefore interpreted by analysing this new local model.

There have not been many studies devoted to the application of XAI methods in a financial context. XAI methods were first applied by Bussmann et al. (2020), Ariza-Garzón et al. (2020), and Gramegna and Giudici (2021). Bussmann et al. (2020) applied a Shapley cost-based XAI model in the context of credit decision-making when small and medium enterprises (SMEs) seek financing through peer-to-peer (P2P) platforms. Ariza-Garzón et al. (2020) analysed the predictive ability of several ML models in the context of credit scoring on P2P lending platforms and used the Shapley method to ensure the explicability of the prediction. Gramegna and Giudici (2021) sought to compare the SHAP and LIME methods by evaluating their ability to identify individual groups of observations using weights assigned to objects using their local interpretability algorithm as an input space for unsupervised and supervised approaches. They used the XGBoost algorithm to predict the probability of default of Italian SMEs.

## Examples of Multi-Criteria Decision-Making in Finance

### Portfolio Optimisation

Portfolio optimisation is an important issue in finance. The goal of portfolio optimisation is to find an effective frontier that shows the highest expected return at each level of portfolio variance. The problem has several objectives and a large decision space. The financial decision-making process is usually based on a choice of promising assets and the allocation of funds among them. The quadratic optimisation problem of maximising expected returns and minimising risk is formulated in modern portfolio theory.

Table 1 lists some examples of studies in which the multi-objective optimisation was applied to the portfolio optimisation problem.

**Table 1:** The most commonly used methods in multi-objective portfolio optimization.

**Method**	**References**	**Frequency of usage/for portfolio optimization**
GP: Genetic Programming	Berutich et al. (2016)	686074/4697
GA: Genetic algorithm	Silva et al. (2015)	293753/4138
GA: Genetic algorithm with Fuzzy Programming	Zhang and Liu (2014); Liu and Zhang (2015); Vercher and Bermúdez (2015)	24293/1263
MDRS: Mean Downside Risk-Skewness	Saborido et al. (2016)	3677/925
MODE: Fuzzy Multi-objective differential Evolution/Fuzzy MOES: Fuzzy Multi-Objective Evolution Strategy/Fuzzy	Pai (2017)	6046/440; 12217/908
NMOEA/D: Normalised Multi-objective Evolutionary Algorithm based on Decomposition	Qu et al. (2017)	4239/312
NSGA II: MOEA/D Multi-objective evolutionary algorithm based on decomposition; Non-dominated sorting genetic algorithm II; GWASF-GA Global Weighting Achievement Scalarizing Function Genetic Algorithm	Meghwani and Thakur (2018)	1061/71; 11533/556; 240/47
SR-MOPSO: Self-regulating multi-objective particle swarm optimization	Mishra et al. (2016)	1683/173
Immunological algorithm	Li and Bao (2014)	28086/133
MOPSO: Multi-objective particle swarm optimization	Babaei et al. (2015); Chen and Zhou (2018)	2558/114
M-CABC: Artificial Bee Colony Algorithm based on Multi-objective covariance	Kumar and Mishra (2017)	506/61
NSGA II and SPEA 2: Strength Pareto evolutionary algorithm 2 and Non-dominated sorting genetic algorithm II	Macedo et al. (2017)	638/56

According to Table 1, the most popular optimisation methods in finance are Genetic Programing (and Algorithm).

Meghwani and Thakur (2017) proposed a candidate selection process and approaches to develop an effective portfolio optimisation model within the framework of a multi-objective evolutionary algorithm (MOEA). These approaches can collectively address a larger category of constraints, i.e., round-lot constraints, cardinality, pre-assignment, budget, and quantities (floor and ceiling). These methods can also be easily integrated into existing evolutionary algorithms (Meghwani and Thakur, 2017). The methods solve multi-objective problems either by combining the objectives into a single objective or by taking only one goal as the objective and turning the others into constraints. Some examples of such objectives are minimising risk, maximising return, characterising uncertainty in terms of the degree of value at risk (VaR) and conditional value at risk (CVaR), and minimising transaction costs. Multi-objective optimization problems are solved using hybrid algorithms such as fuzzy, GA, MOES, and NSGA II algorithms (Milhomem and Dantas, 2020).

### Pension Fund Evaluation

Numerous methods are used to evaluate pension funds. Table 2 lists some of the most commonly used methods.

**Table 2:** Methods most commonly used in pension fund evaluation.

**Method**	**References**	**Frequency of usage/for pension funds**
Stochastic dominance (SD)	Kopa (2016), Moriggia et al. (2019), Kabašinskas et al. (2020)	25225/84
Sharpe ratio, Jensen's alpha, beta or Treynor indicators	Shah and Hijazi (2005), Jagric et al. (2007), Bohl et al. (2011), HemaDivya (2012), Aygoren et al. (2017), Mestan et al. (2017)	66961/31
Sortino index, Fama index, Sterling indicator	Hribernik and Vek (2011), Kolbadi and Ahmadinia (2011), Prajapati and Patel (2012), Parlak (2014), Kupčík and Gottwald (2016)	21428/1303
Capital Asset Pricing Model (CAMP)	Bohl et al. (2011), Adami et al. (2014)	2213/ 200
A multistage risk-averse stochastic optimization model	Kabašinskas et al. (2019), Moriggia et al. (2019)	719/43
Analytic Hierarchy Process (AHP)	Voronova (2011)	171945/811
TOPSIS	Imam and Gurol (2018)	8587/13

According to Table 2, various indicators (Sortino, Sharpe, Fama etc) are the most popular portfolio (pension fund) evaluation methods. Moreover, they are also used in decision-making when Analytic Hierarchy Process (the most popular DM after optimisation) and other MCDMs are applied.

### Bankruptcy Prediction and Credit Risk Assessment

The main problems that are evaluated by MCDM in the area of finance are fraud risk, bankruptcy prediction, credit risk assessment and prediction, loan default prediction, and business failure prediction. Predicting bankruptcy has long been an important problem in the field of finance and management and has attracted the attention of many researchers and businesses. Table 3 summarises the main methods used in bankruptcy prediction and credit risk assessment.

**Table 3:** The most commonly used methods in bankruptcy prediction and credit risk assessment.

**Method**	**References**	**Frequency of usage/bankruptcy or credit risk**
PROMETHEE	Chen and Hu (2011); Peng et al. (2011); Vukovic et al. (2012); Doumpos and Zopounidis (2013)	3313/35
ELECTRE	Hu (2009), Li and Sun (2009), Gastelum-Chavira et al. (2017)	3777/49
VIKOR	Yalcin et al. (2012), Alvandi et al. (2013), Farrokh et al. (2016)	2671/22
TOPSIS	Secme et al. (2009), Garc'ia et al. (2010), Mandic et al. (2014), Wanke et al. (2016), Ignatius et al. (2018)	8587/64
Artificial Neural Network (ANN)	Bequé et al. (2017), Son et al. (2019), Tumpach et al. (2020)	177505/564
Support Vector Machine (SVM)	Barboza et al. (2017), Liang et al. (2018), Sun et al. (2018), Feng et al. (2018), Lin et al. (2019), Ribeiro et al. (2019)	214725/387
Logistic Regression (LR)	Bequé et al. (2017), du Jardin (2017), Zelenkov et al. (2017), Chen and Zhou (2018), Lin et al. (2019)	104865/1315
Decision Tree (DT)	Zelenkov et al. (2017), Feng et al. (2018)	259019/1176

As it can be seen from Table 3, Logistic Regression is the most popular method when bankruptcy or credit risk is evaluated. It is followed by Decision Trees, Artificial Neural Networks and Support Vector Machines. TOPSIS is the most popular MCDM in this field.

It is impossible to specify any one of these methods that will always be the most suitable and work better than any other (Wang et al., 2018). There are a number of methods and strategies, both individual and hybrid, for predicting financial difficulties, and these different methods and strategies perform differently in different situations, depending on the data and variables involved.

## Discussion

Zopounidis (1999) noted that there are three main steps in multi-criteria decision-making: problem specification, model development, and decision-making. Zopoundis recommended that investment decisions be made using typical information acquired from standard sources (financial statements, CAPM, etc.), which was sufficient two decades ago and could be done using calculations performed with Microsoft Excel. However, the volume of available financial information increased more than a hundredfold during this period, up to 64.2 zettabytes in 2020 (Statista, 2021). In addition, data structures became more complex, and extraction of useful information became more challenging. A big data framework may be a partial solution to this problem. However, computing and human power are not sufficient to philtre, cluster, and explain all of the important investment information available. AI offers a potential way to overcome this limitation.

Tables 1–3 show that the choice of a particular multi-criteria approach is strongly influenced not only by the problem it is trying to solve, but also by the amount of data involved and the criteria of interest. The most commonly used methods for predicting credit risk and bankruptcy are decision trees and logistic regression, which are also easy to explain. The use of neural networks, often referred to as “black box” methods, usually provides the best estimate of the quality of the study and offers higher predictive accuracy than the most commonly used methods. The implementation and development of trustworthy AI-based methods is becoming increasingly important, and thus XAI is becoming a key component of machine learning.

AI and machine learning have been applied to many problems in finance, ranging from task automation to chatbot assistants to fraud detection. AI is quickly reshaping the financial industry. To eliminate cultural prejudices and focus on the most pressing security issues, it is important to promote truthful, rational discourse. It becomes a great challenge when using AI techniques to comply with all recommendations while maintaining an appropriate level of interpretability and high accuracy. Transparency and explainability are critical issues for policymakers and regulators. This is particularly obvious in the financial and banking sectors, where AI use has expanded to risk management, predictive analytics, and fraud detection. Legal frameworks should be adapted to take into account the risks and potential of new technologies. AI developers must be obliged to invest more in ensuring the integrity and accuracy of these technologies. The main concerns, such as predictability, transparency, explainability, and non-manipulability should be emphasised.

Among the most important concerns associated with using AI techniques in finance are transparency and interpretability. The ability to create artificial systems causes ethical problems that are indistinct from natural conscious individuals and therefore also potentially conscious. First of all, during the transition from the standard AI to the expanded intelligence, systems acquire the ability to connect new program procedures. Since such systems follow this line of execution, they need to be handled cautiously and experimented with in a closed context. With the right choice of embodiment in a virtual machine or in a robotic body, a person should be able to solve such problems (Krauss and Maier, 2020). The more we approach human behaviour, the more other ethical problems arise. The ability to reproduce and copy the same body and mind does not further alleviate the problem and implies that we need to agree on the ethics and standards of AI in the near future.

The growing attention paid to AI by governments shows that AI is having an increasing impact on the lives of people and businesses. This is reflected in the various funded projects on AI. Among these is the Horizon Europe research and innovation funding program, which will continue until 2027. The main objective of the European Partnership on Artificial Intelligence, Data and Robotics is to bring the greatest benefit to Europe from AI, data, and robotics. This cooperation will stimulate innovation and adoption of these technologies. The partnership is expected to drive new markets, software applications, and investments that will create technical, economic, and social value for businesses, citizens, and the environment. By 2030, the EU is expected to commit to the development and implementation of robust, secure, and trustworthy AI, data, and robotics that are compatible with EU values and regulations. AI is also an important topic in Project DNA for Lithuania.

A promising area of future research in the field of finance is the development of MCDM models that use AI methods to improve the reliability and accuracy of models for solving financial problems that can be applied in practise.

## Conclusions

There are many different models and validation methods available to aid in financial data mining and decision-making. It is difficult to determine whether any of these methods is superior to the others. It may be more beneficial to take advantage of the strengths of different methods and combine them to make more informed financial decisions. The main advantages of the MCDM principle in financial decision-making are the ability to structure complex evaluation tasks that allow for well-founded financial decisions, the application of quantitative and qualitative criteria in the analysis process, the possibility of transparency of evaluation, and the introduction of improved, universal, and practical academic methods to the financial decision-making process. The main purpose of MCDM is to provide a set of integration mechanisms that allow for the development of decision support models based on system preferences and adjudication policy.

**Figure 6:**
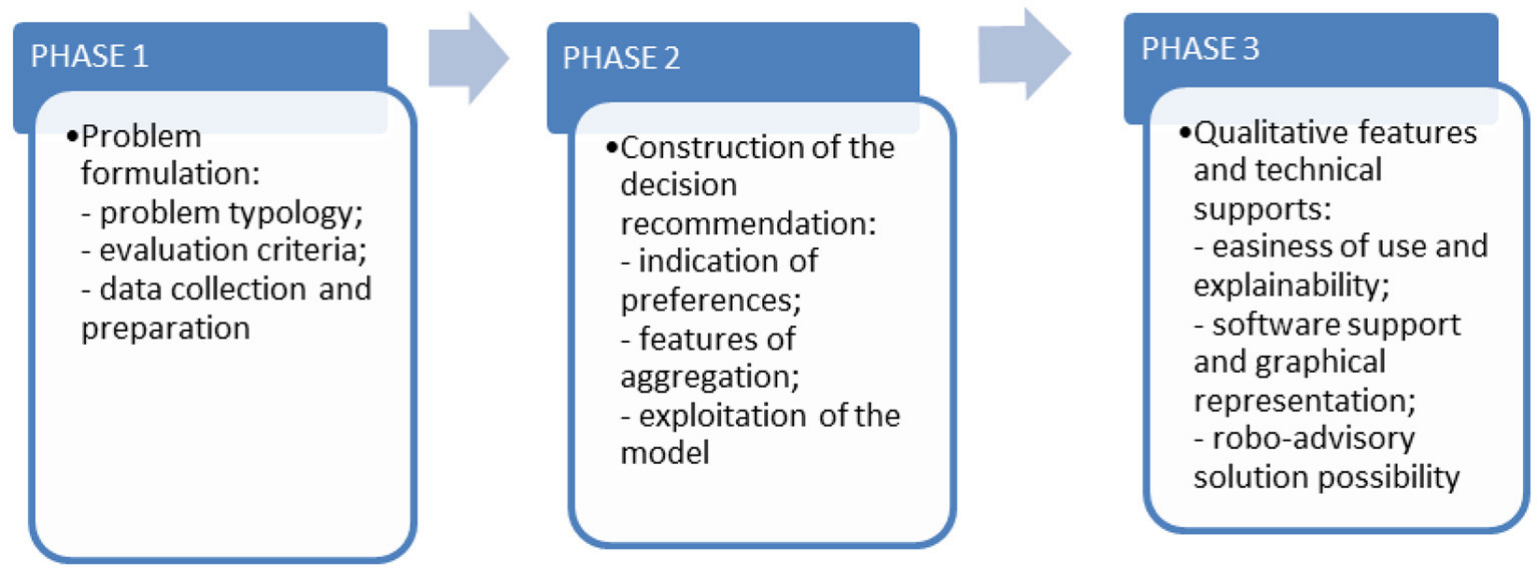
Proposed MCDM process to be developed in future research.

Future research in this area should include the machine learning method as well as AI and MCDM models to provide practical solutions to the most complex problems. Taking into account the examples analysed and the discussion presented in this article, we suggest an updated MCDM process illustrated in Figure 6.

Decision-making support is a complex task because it requires a large number of skills, including problem-solving, programming, statistical modelling, project planning, project management, and risk assessment. The MCDM process can be made more manageable by divided into the following three phases: problem formulation, construction of the decision recommendation, and provision of qualitative technical support features.

Compared to AI methods, MCDMs are transparent decision-making tools. As MCDM and AI methods are usually used to solve multi-criteria problems on a comparative basis (to see which method yields better results), the development of hybrid multi-criteria decision-making methods (MCDM + AI) could be consistent with the principles of XAI development.

The degree to which one person can understand the reason for a decision or predict the result that a model will produce is called interpretability. Thus, when choosing a research model, it is necessary to select a method that yields result that not only are the best possible technical solutions but also are easy to interpret.

## Author Contributions

AK and JČ: conceptualisation. JČ: writing-original draft preparation and visualisation. AK: writing-review and editing, supervision, and funding acquisition. Both authors contributed to the article and approved the submitted version.

## Funding

The research of AK was partially supported by COST (European Cooperation in Science and Technology) Action 19130: Fintech and Artificial Intelligence in Finance – Towards a transparent financial industry (FinAI).

## Conflict of Interest

The authors declare that the research was conducted in the absence of any commercial or financial relationships that could be construed as a potential conflict of interest.

## Publisher's Note

All claims expressed in this article are solely those of the authors and do not necessarily represent those of their affiliated organizations, or those of the publisher, the editors and the reviewers. Any product that may be evaluated in this article, or claim that may be made by its manufacturer, is not guaranteed or endorsed by the publisher.
